# The cruciality of increasing index of suspicion for atypical *Bartonella henselae* in pediatric patients: A case series

**DOI:** 10.1016/j.idcr.2025.e02192

**Published:** 2025-03-05

**Authors:** Victoria Vazquez, Lorraine Bermudez-Rivera, Arino Neto, Vanessa Perez, Adriana Cadilla, Linette Sande

**Affiliations:** a Nemours Children’s Hospital – Department of Graduate Medical Education, 6535 Nemours Parkway, Orlando, FL 32827, USA; b University of Central Florida College of Medicine, 6850 Lake Nona Blvd, Orlando, FL 32827, USA; c Ann & Robert H Lurie Children’s Hospital of Chicago – Department of Pediatrics Emergency Medicine, 225 E. Chicago Avenue, Box 86, Chicago, IL 60611, USA; d Advent Health, Department of Pediatric Emergency Medicine, 1500 SW 1st Ave, Ocala, FL 34471, USA

**Keywords:** *Bartonella henselae*, Bartonella endocarditis, Bartonella osteomyelitis, Bartonella neuroretinitis, Bartonella encephalitis, Atypical bartonellosis

## Abstract

*Bartonella henselae*, a gram-negative rod, is the etiologic agent of cat scratch disease, which may manifest as a self-limiting local infection or as an atypical, invasive disease. Establishing *B. henselae* as the causative organism can be challenging as it is a fastidious organism that does not grow on traditional media. Diagnosis is generally performed with serology, which has variable turnaround times, or microbial cell-free DNA (mcfDNA) sequencing, which has a high cost. Therefore, a high index of suspicion is necessary for timely diagnosis and management of these invasive infections. We depict a case series of nine atypical Bartonella infections in children. By highlighting these presentations, their diagnostic testing, and treatment, we emphasize the significance of an elevated index of suspicion of atypical bartonellosis for accurate diagnosis and timely antibiotic management. Our invasive Bartonella cases entail culture-negative subacute endocarditis, osteomyelitis, neuroretinitis, encephalitis, hepatosplenic disease, and lymphadenopathy with splenic involvement.

## Introduction

*Bartonella henselae*, a gram-negative rod, is the etiologic agent of cat scratch disease and is primarily seen in school-aged children [1], [2]. Bartonellosis is usually a self-limiting disease in immunocompetent patients, commonly manifesting as a local infection (e.g., lymphadenopathy) and less often as an invasive disease [3].

Awareness of atypical presentations is crucial, as serology turnaround time may delay the diagnosis. Although not always present, cat exposure history should be elicited. Furthermore, once *Bartonella henselae* is considered, establishing it as the causative organism can be challenging because it does not grow on traditional media [4]. Despite its variable turnaround time, sensitivity, and specificity, serologic testing is currently the primary method used to diagnose *B. henselae* [1], [4]. Testing can also be obtained by conventional PCR tests and DNA detection in biopsy samples; however, a biopsy is invasive [1], [4], [5]. Microbial cell-free DNA (mcfDNA) sequencing performed by next-generation sequencing (NGS) may offer a quicker diagnosis, although at a higher cost [5], [6].

Treatment of bartonellosis varies depending on clinical presentation. While most immunocompetent patients do not require treatment, timely antibiotic therapy is essential in atypical presentations [5], [7]. Given the indication of opportune antibiotic treatment and the variable turnaround time of diagnostic testing, bartonellosis must be strongly suspected so that appropriate therapy can be started promptly.

We depict a case series of nine atypical pediatric presentations of invasive bartonellosis at our institution from 2017 to 2023 (Table 1). Our hospital’s institutional board review (IRB) considered this study exempt. Our objective is to highlight these unusual presentations and the strong clinical suspicion required for diagnosing and treating these atypical cases.

**Table 1:** Overview of nine atypical presentations, confirmatory testing, and treatment of atypical invasive bartonellosis.

Case Number	Admitting Chief Complaint	Invasive *B. henselae* diagnosis	Confirmatory Testing & Hospital Day (HD) Ordered	Turnaround Time	Antibiotic Treatment &HD Started & Length of Treatment	*B. henselae* Initial Serology Results
1	Shortness of breath with exertion	Subacute culture negative endocarditis	Serology HD 2 Microbial cell free DNA (mcfDNA) HD 7	5 days 2 days	Gentamicin HD 2 6 weeks Ceftriaxone HD 2 4 days Doxycycline HD 7 13 weeks	IgG^1^ 1:2560 IgM^2^ Negative
2	Leg pain and fever	Osteomyelitis	Serology HD 6	3 days	Cefazolin HD 3 5 days Clindamycin HD 5 3 days Azithromycin HD 9 6 weeks Rifampin HD 9 12 weeks Trimethoprim-Sulfamethoxazole (TMP-SMX) 6 weeks after discharge 6 weeks	IgG^1^ 1:1280 IgM^2^ 1:200
3	Weight loss, abdominal pain, and fever	Neuroretinitis	Serology HD 5	5 days	Doxycycline HD 5 7 weeks Rifampin HD 5 7 weeks	IgG^3^ > =1:1024 IgM^4^ > =1:20
4	Arthralgias and emesis	Encephalitis	Serology HD 7 McfDNA HD 4	5 days 2 days	Ceftriaxone HD 1 4 days Doxycycline HD 4 10 days Rifampin HD 4 10 days	IgG^1^ 1:640 IgM^2^ Negative
5	Hip pain and fever	Osteomyelitis	Serology HD 5	3 days	Azithromycin HD 5 10 days^5^ Rifampin HD 5 10 days^5^	IgG^3^ > =1:1024 IgM^4^ < 1:20
6	Fever of unknown origin	Hepatosplenic bartonellosis	Serology HD 7	6 days	Azithromycin HD 1 24 days Rifampin HD 5 14 days	IgG^1^ 1:1280 IgM^2^ Negative
7	Axillary abscess	Lymphadenopathy and splenic bartonellosis	Serology HD 4	4 days	TMP/SMX HD 1 10 days	IgG^1^ 1:1280 IgM^2^ Negative
8	Weight loss, loss of appetite, and fatigue	Subacute culture negative endocarditis	Serology 8 days after hospital discharge	4 days	Vancomycin HD 1 1 month Ceftriaxone HD 1 1 month Doxycycline HD 17 33 months Gentamicin Outpatient day 14 18 days	IgG^3^ > =1:1024 IgM^4^ > =1:20
9	Right leg pain and limping	Osteomyelitis	Serology 1 day after hospital discharge	6 days	Cefazolin HD 3 4 days Rifampin HD 5 6 weeks TMP/SMX HD 5 6 weeks	IgG^1^ 1:1280 IgM^2^ Negative

1. Negative = <1:320 titer

2. Negative = <1:100 titer

3. Negative = <1:128 titer

4. Negative = <1:20 titer

5. Patient lost to follow-up

## Case 1

A 17-year-old female with history of tetralogy of Fallot status post complete repair, fetal alcoholic syndrome, and epilepsy was admitted for three weeks of dyspnea on exertion, fatigue, and weight loss. Her echocardiogram noted two masses on the pulmonary valve. Laboratory workup showed pancytopenia, elevated C-reactive protein (CRP), and glomerulonephritis. Blood cultures remained negative. Given the patient's presentation of culture-negative subacute endocarditis and cat exposure, bartonellosis was suspected. *Bartonella* serology was obtained, and she was started on gentamicin and ceftriaxone for empiric treatment of endocarditis. Plasma mcfDNA was also sent and resulted positive for *B. henselae*. Serology was positive for *B. henselae* IgG titer > 1:2560 (normal <1:320) with a negative IgM titer. The patient was transitioned to doxycycline and continued on gentamicin. She had clinical improvement and completed 6 weeks of gentamicin and 13 weeks of doxycycline. Repeat serology showed *B.henselae* IgG titer 1:2560 (normal <1:320) with a negative IgM titer.

## Case 2

A 4-year-old healthy male came to the emergency department (ED) due to six days of right knee pain and fever. Laboratory workup demonstrated elevated CRP and erythrocyte sedimentation rate (ESR). Right hip and knee ultrasound were unremarkable. MRI of the right hip to ankle was significant for osteomyelitis of L5 with a questionable early intraosseous abscess. Cefazolin was started. Due to increasing CRP, vertebral osteomyelitis, and lack of clinical improvement, *Bartonella* serology and plasma mcfDNA were ordered. He was switched to clindamycin without improvement. The mcfDNA test was negative. Serology was positive for *B. henselae* IgG titer 1:1280 (normal <1:320) and IgM titer 1:200 (normal <1:100). Patient had a young dog and remote cat exposure. The antibiotic regimen was switched to rifampin and azithromycin. He improved clinically and was treated with 6 weeks of azithromycin and rifampin. He was later changed to trimethoprim/sulfamethoxazole (TMP/SMX) plus rifampin for 6 weeks due to increasing ESR, with subsequent normalization of ESR. Repeat serology showed *B. henselae* IgG titer 1:640 (normal <1:320) and negative IgM.

## Case 3

A healthy 16-year-old female presented with a four-week history of weight loss, abdominal pain, and fever. In the ED, she had an unremarkable CT of the abdomen and pelvis, CT of the brain, and laboratory workup. Further history revealed she had pet birds, dogs, and cats. *Bartonella* serology was obtained. She continued having intermittent fevers, headaches, blurry vision, and vomiting. She was treated for an intractable migraine without improvement. On day five of admission, serology was positive for *B. henselae* IgG titer > =1:1024 (normal <1:128) and IgM titer > =1:20 (normal <1:20). She was started on doxycycline and rifampin for suspected neuroretinitis. The initial eye exam did not note neuroretinitis, and ophthalmology did not recommend corticosteroids. MRI orbit and MRV head were both unremarkable. She was discharged on day five of admission with a diagnosis of suspected *B. henselae* neuroretinitis. The outpatient follow-up eye exam was consistent with neuroretinitis; however, no corticosteroids were started since the patient had improved vision. She continued to improve with seven weeks of rifampin and doxycycline. Repeat serologies were not obtained.

## Case 4

A healthy 9-year-old male was admitted to an outside facility with status epilepticus. A broad differential was considered. Cerebrospinal fluid (CSF) studies were unremarkable, including a negative CSF meningitis panel (included *E. coli K1*, *H. influenzae, Listeria monocytogenes*, *N. meningitidis, S. agalactiae, S. pneumoniae*, Cytomegalovirus (CMV), Enterovirus, Herpes simplex virus 1 (HSV-1), Herpes simplex virus 2 (HSV-2), Human Herpes Virus 6 (HHV-6), Human Parechovirus, Varicella zoster virus*,* and *Cryptococcus* (C. *neoformans/C. gattii).* CSF West Nile antibody and blood HSV PCR were negative. Head CT was unremarkable. He was transferred to our facility for further management. Video electroencephalogram showed subclinical seizures. A brain MRI was normal. Plasma mcfDNA test and *Bartonella* serology were collected given the patient's lack of clinical improvement and recent history of a kitten scratch (although the timing was unknown). Ceftriaxone was continued for empiric meningitis coverage until CSF culture was negative at 48 hours. The mcfDNA test and serology were positive for *B. henselae,* with IgG titer 1:640 (normal <1:320) and negative IgM titer. He was transitioned to doxycycline and rifampin. He showed clinical improvement and was discharged home to complete a 10-day course of doxycycline and rifampin. Repeat serologies were not obtained.

## Case 5

A healthy 9-year-old male was admitted for fever and left hip pain. His symptoms began one month prior to presentation when he was diagnosed with Group A *Streptococcus* pharyngitis and influenza. After no improvement, he went to an outside ED for evaluation. He was febrile with severe hip pain. He had elevated CRP and an unremarkable hip x-ray. He was transferred to our hospital for further management. He had a painful, left-sided limp. Laboratory workup was significant for leukopenia and elevated CRP and ESR. MRI of bilateral hips to ankles showed multifocal bony lesions concerning for an oncologic process. The patient underwent joint aspiration and bone marrow biopsy, which were negative for malignancy. Given the patient's lack of clinical improvement, history of cat exposure, and negative oncologic workup, *Bartonella* was suspected, and serology was collected. *B. henselae* IgG titer was > = 1:1024 (normal <1:128) and IgM titer < 1:20 (normal <1:20). He was diagnosed with likely *Bartonella* osteomyelitis and had clinical improvement with azithromycin and rifampin at time of discharge (patient was lost to follow-up).

## Case 6

**Fig. 1:**
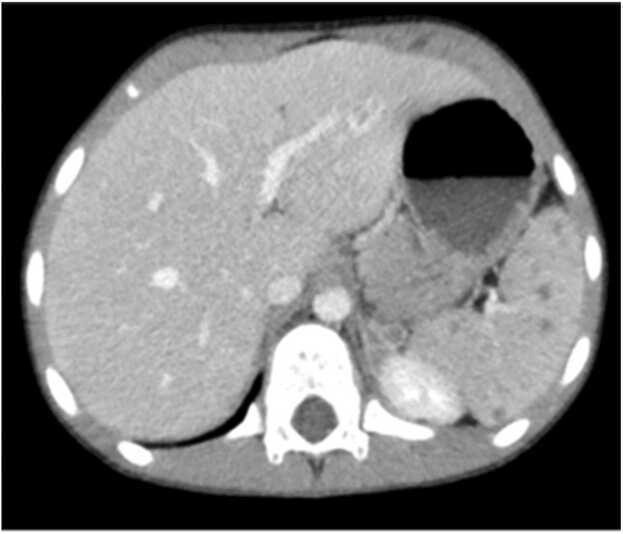
Case 6. CT abdomen with innumerable small hypoattenuating foci throughout the liver and spleen.

**Fig. 2:**
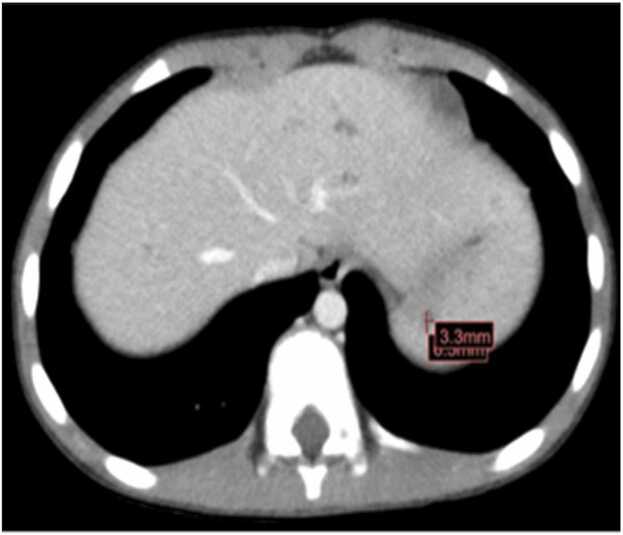
Case 6. CT abdomen with innumerable small hypoattenuating foci throughout the spleen.

A healthy 8-year-old female was admitted with one month of intermittent fevers and malaise. She had been clinically diagnosed with a urinary tract infection 3 weeks prior and treated with two courses of cephalexin. Despite this, she continued to have worsening malaise and fevers, as well as a new cough with nasal congestion. She presented to an outside ED, where an abdominal ultrasound noted multiple hypoechoic areas throughout the spleen and a CT abdomen, which showed numerous small hepatosplenic lesions (Fig. 1, Fig. 2). She had an elevated WBC, ESR, and CRP, a respiratory viral panel positive for human rhinovirus/enterovirus, and a urinalysis with moderate WBCs and 3 + leukocyte esterase. She received one dose of ceftriaxone and was transferred to our hospital for further management. She had cervical and axillary lymphadenopathy with no palpable hepatosplenomegaly. Hepatitis panel, CMV serology, and *Toxoplasma gondii* antibody returned negative. Given the hepatosplenic lesions, lymphadenopathy, and history of cat exposure, *B. henselae* was suspected, and serologies were collected. She was empirically started on azithromycin. Blood cultures remained negative; however, she continued to have fevers and up-trending CRP. Rifampin was started, in addition to azithromycin, for suspected hepatosplenic bartonellosis. Serology showed a positive *B. henselae* IgG 1:1280 (normal <1:320) with a negative IgM titer. She improved clinically and was discharged with 10 additional days of azithromycin and rifampin. As an outpatient, an additional 7 days of azithromycin were prescribed. Repeat serology showed *B. henselae* IgG titer 1:512 (normal <1:64) and negative IgM titer.

## Case 7

**Fig. 3:**
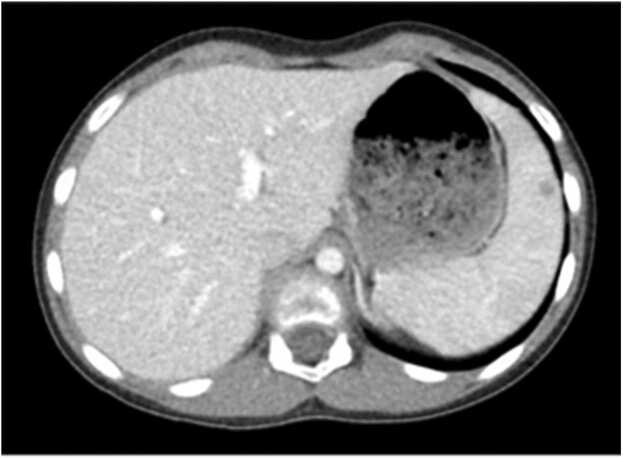
Case 7. CT thorax/abdomen with small nonspecific low-attenuation lesions in the spleen.

**Fig. 4:**
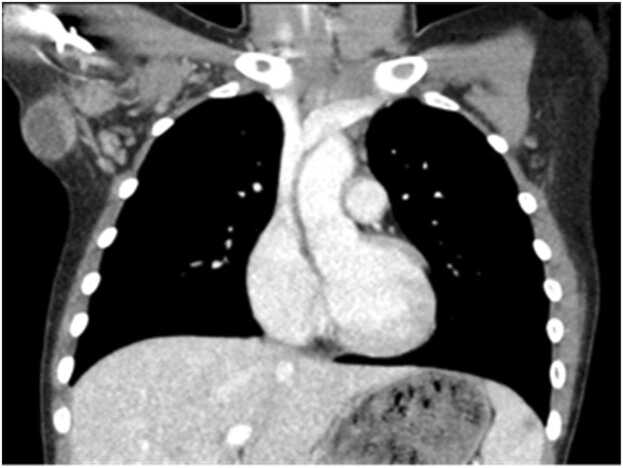
Case 7. CT thorax/abdomen with peripherally enhancing bilobed fluid collection in the right axilla.

A 9-year-old female with recent history of immune thrombocytopenic purpura (ITP) was admitted for worsening axillary cellulitis refractory to multiple antibiotics, including azithromycin. She reported having three cats but no scratches. An axillary ultrasound noted a 4.8 cm abscess. A follow-up chest CT showed a peripherally enhancing bilobed fluid collection in the right axilla with inflammatory changes, two small non-specific low attenuation lesions in the spleen, and splenomegaly (Fig. 3, Fig. 4). She had axillary lymphadenopathy on physical exam without palpable hepatosplenomegaly. *Bartonella* serology was sent, and she was started on TMP/SMX for suspected *B*. *henselae* infection. A needle aspiration was considered, but given suspected *Bartonella* infection, antibiotic therapy was trialed first. She was transitioned to oral TMP/SMX and discharged. Serology showed a positive *B. henselae* IgG titer of 1:1280 (normal <1:320) with a negative IgM titer. She had clinical improvement with the completion of a 10-day course of TMP/SMX and followed up with her pediatrician for abscess resolution.

## Case 8

A 13-year-old male with history of thoracoabdominal coarctation of aorta status post repair, bicuspid aortic valve, and renal artery stenosis was admitted to an outside hospital with eleven months of worsening weight loss, decreased appetite, and fatigue. Echocardiogram was notable for a vegetation on the posterior aortic valve leaflet, as well as thickened and irregular aortic bypass graft. On admission, the patient was febrile, anemic, and found to have elevated ESR and CRP. Blood cultures were obtained, and he was started on vancomycin and ceftriaxone for subacute endocarditis. He was transferred to our facility for further management.

He had a systolic ejection murmur. *Bartonella* species PCR from blood and *Brucella* serology were negative. He was placed on vancomycin, ceftriaxone, and doxycycline for culture-negative endocarditis. Despite his antibiotic regimen, he continued having chest pain and elevated blood pressures as an outpatient. However, the vegetation on his echocardiogram remained stable. Given his continued symptoms, history of cat exposure, and negative blood cultures, *Bartonella* serology was obtained. Serology showed *B. henselae* IgG > =1:1024 (normal <1:128) with IgM titer > =1:20 (normal <1:20) and *B. quintana* IgG titer > =1:1024 (normal <1:128) with a negative IgM titer. He received 2 weeks of gentamicin, as well as a protracted course of doxycycline based on his underlying cardiac condition, persistent vegetation, and fluctuating *Bartonella* titers. He was followed for 3 years, and his titers were trended until the resolution of his symptoms.

## Case 9

A 3-year-old under-immunized male was admitted with four weeks of intermittent fever and a right-sided limp. The patient was born in Chile and moved to the United States two years prior. He was seen at an outside ED, where his initial workup was significant for leukocytosis and elevated CRP. A neck ultrasound demonstrated prominent lymph nodes bilaterally. X-rays of the right femur and tibia/fibula were unremarkable. He was transferred to our hospital for further management.

Due to the presence of cervical lymphadenopathy, Ebstein-Barr virus (EBV), CMV, and *Bartonella* serologies were obtained. MRI of the right lower extremity was significant for multiple foci of abnormal bone marrow edema in the pelvic bones, concerning for osteomyelitis versus Ewing sarcoma. He underwent a diagnostic bone biopsy, which did not show any signs of malignancy. He was started on cefazolin for suspected bacterial osteomyelitis. Given diagnostic uncertainty and continued elevated CRP, he was also tested for malaria, tuberculosis, and *Brucella*, although he had no history of cat, dog, or farm animal exposure. *Brucella* IgM was positive. Antibiotics were transitioned to rifampin and TMP/SMX and he was discharged with a diagnosis of *Brucella* osteomyelitis. *Bartonella* serology was pending at discharge. Following discharge, *Brucella* confirmatory antigen resulted negative, and *Bartonella* serology showed *B.henselae* IgG 1:1280 (normal <1:320) with a negative IgM titer. He remained afebrile and had improvement in his limp with a six-week course of rifampin and TMP/SMX to treat suspected *Bartonella* osteomyelitis. Repeat titers were not obtained.

## Discussion

Our case series presents nine atypical bartonellosis presentations. Atypical presentations of *B. henselae* are rare in immunocompetent patients [8], [9]. Without treatment, atypical bartonellosis may be associated with a high rate of morbidity, emphasizing the need to start treatment promptly; however, the role of antibiotics is still unclear for many bartonellosis presentations [7], [17]. Given that all nine cases ranged in severity of presentation, it is difficult to compare outcomes based on the timing of treatment; however, all nine patients improved after their antibiotic regimen targeted a *Bartonella henselae* infection.

Bartonellosis is not often considered in the initial differential diagnosis for patients presenting with an atypical infection. Serologic testing is the primary method of diagnosis; however, given its variable turnaround time and sensitivity, a strong clinical suspicion is required to ensure timely treatment [1], [4]. Therefore, clinicians often initiate antibiotic treatment before the results of serologic testing [1], [4]. Serology was ordered for all nine patients, but only six received appropriate antibiotic therapy before the results were available, further emphasizing the utility of a high index of suspicion in ensuring timely treatment.

Testing for *B. henselae can* also be obtained by DNA detection through biopsy and a mcfDNA sequencing test, such as a Karius test [4], [6]. McfDNA testing has been demonstrated to have a higher yield than blood culture in identifying pathogens in osteoarticular infections [18], [19].

A distinct difference between mcfDNA and *Bartonella* serology is that the latter is known to have cross-reactivity with organisms, such as *Bartonella quintana*, *Coxiella burnetii*, and Chlamydia species [5], [6]. In Case 9, cross-reactivity led the patient to have an incorrect diagnosis of *Brucella* osteomyelitis. Nonetheless, medical management did not change when the correct diagnosis was made, given the similarities in treatment. However, *Bartonella* cross-reactions with other organisms, such as *Coxiella*, are essential to identify. Patients may present with similar symptoms, but the treatment regimens are distinct [7], [13], [14]. In Case 2, the mcfDNA was negative, and diagnosis was made through serology. To aid in the diagnosis of patients where there is a continued index of suspicion, Bartonella serology should still be obtained.

Bartonella endocarditis has a significantly higher rate of mortality when compared to endocarditis caused by other pathogens [7], [15]. Diagnosis is often challenging as *Bartonella* does not grow on standard culture. Therefore, awareness of this condition is necessary to initiate appropriate management [7]. According to the revised 2023 Duke-International Society for Cardiovascular Infectious Diseases Criteria for Infective Endocarditis, Case 1 and Case 8 presentations fulfill the criteria for endocarditis. These infective endocarditis guidelines recently recognized Bartonella as a significant causative organism for endocarditis [16]. This revision of the guidelines acknowledges the difficulty in isolating Bartonella in culture. It highlights that newer technology, such as metagenomics, can play a pivotal role in reaching an accurate diagnosis.

Neuroretinitis is a rare ocular manifestation of *Bartonella henselae. Although* the use of antibiotics in *Bartonella* neuroretinitis remains controversial, it has been concluded that antibiotic therapy shortens the course of illness and improves the timing of return for visual acuity [10]. Therefore, awareness of this complication is essential to ensure the timely return of visual acuity.

*Bartonella* testing is rarely obtained among patients with encephalitis; therefore, clinical suspicion is required for a prompt diagnosis [11], [12]. Given the unknown etiology of the patient's encephalitis in Case 4, a mcfDNA test was sent early in the hospital course, allowing the patient to be transitioned to appropriate antibiotics soon after diagnosis.

## Conclusion

Our case series highlights the urgency of having a high index of suspicion for atypical *B. henselae* in the pediatric population. Consideration of early antibiotic treatment for atypical bartonellosis, especially with a cat exposure history, is crucial to improve patient outcomes and avoid unnecessary invasive procedures. Challenges in serologic testing may delay diagnosis; microbial cell-free DNA sequencing may provide quicker identification and treatment, though cost is a limitation. Our case series underscores diverse presentations of *B. henselae*, emphasizing the cruciality of timely diagnosis, a high index of suspicion, and prompt treatment for improved outcomes.

## Funding

This research did not receive any specific grant from funding agencies in the public, commercial, or not-for-profit sectors.

## CRediT authorship contribution statement

**Sande Linette:** Writing – review & editing, Supervision, Methodology, Conceptualization. **Cadilla Adriana:** Writing – review & editing, Supervision, Methodology, Conceptualization. **Vazquez Victoria:** Writing – review & editing, Writing – original draft, Resources, Methodology, Investigation, Formal analysis, Data curation, Conceptualization. **Perez Vanessa:** Writing – review & editing, Supervision, Methodology, Conceptualization. **Neto Arino:** Visualization, Methodology, Data curation, Conceptualization. **Bermudez-Rivera Lorraine:** Project administration, Methodology, Formal analysis, Data curation.

## Declaration of Competing Interest

The authors declare that they have no known competing financial interests or personal relationships that could have appeared to influence the work reported in this paper.
